# Evaluation of markers of outcome in real-world treatment of diabetic macular edema

**DOI:** 10.1186/s40662-018-0119-9

**Published:** 2018-10-11

**Authors:** António Campos, Elisa J Campos, Anália do Carmo, Francisco Caramelo, João Martins, João P Sousa, António Francisco Ambrósio, Rufino Silva

**Affiliations:** 1 Coimbra Institute for Clinical and Biomedical Research (iCBR), Faculty of Medicine, University of Coimbra, Azinhaga de Santa Comba, Celas, 3000-548 Coimbra, Portugal; 2 CNC.iCBR Consortium, University of Coimbra, Coimbra, Portugal; 3 Department of Ophthalmology, Leiria Hospital, Leiria, Portugal; 4 Clinical Pathology Department, Centro Hospitalar Universitário de Coimbra (CHUC), Coimbra, Portugal; 5 Laboratory of Biostatistics and Medical Informatics, Faculty of Medicine, University of Coimbra, Coimbra, Portugal; 6 Medical Sciences Department, Faculty of Health Sciences, University of Beira Interior, Covilhã, Portugal; 7 Association for Innovation and Biomedical Research on Light and Image (AIBILI), Coimbra, Portugal; 8 Department of Ophthalmology, Centro Hospitalar Universitário de Coimbra (CHUC), Coimbra, Portugal

**Keywords:** Diabetic macular edema, Outcome factors, Spectral-domain optical coherence tomography, Anti-vascular endothelial growth factor, Laser

## Abstract

**Objective:**

To evaluate short-term markers of outcome in diabetic macular edema (DME).

**Methods:**

Prospective interventional case series included 122 eyes of 122 patients with recently diagnosed DME. Eyes were treated with a 3-monthly loading dose of ranibizumab or aflibercept and pro re nata thereafter. Serial enhanced deep imaging SD-OCT high resolution scans were used to measure subfoveal choroidal thickness (SFCT) and central retinal thickness (CRT). Anatomic (10% CRT decrease) and functional responses (best corrected visual acuity, BCVA gain ≥5 letters) were assessed at 3 months and 6 months using univariate and multivariate analyses. Parameters tested were gender, duration of diabetes, HbA1c, hypertension, CRT, SFCT, BCVA, ellipsoid zone (EZ) status, subfoveal neuroretinal detachment (SND), anti-VEGF used and laser naivety. A logistic regression model was applied to find independent markers outcome.

**Results:**

BCVA increased, CRT and SFCT decreased at 3 months and 6 months. Good metabolic control (*p* = 0.003), intact baseline EZ (*p* = 0.030), EZ re-grading at 3 M (*p* < 0.001) and laser naivety (*p* = 0.001) were associated with better functional outcome. The multivariate linear regression model showed that baseline SND and CRT are predictors of anatomic response, while lower baseline BCVA and intact EZ are predictors of functional response.

**Conclusion:**

The presence of SND predicts anatomic response only, while an intact EZ is critical to achieve a good functional outcome in DME.

**Electronic supplementary material:**

The online version of this article (10.1186/s40662-018-0119-9) contains supplementary material, which is available to authorized users.

## Background

Diabetic macular edema (DME) is the leading cause of blindness in patients with diabetic retinopathy worldwide [1]. Blood retinal barrier dysfunction, inflammation and choroidopathy seem to contribute to DME pathogenesis [2]. Optical coherence tomography (OCT) became the most useful tool for the evaluation and follow-up of DME and enhanced deep imaging spectral domain optical coherence tomography (EDI SD-OCT) was successfully used in evaluating choroidal thickness [2]. Treatment of DME shifted from laser photocoagulation to anti-VEGF therapy. However, DME exhibits wide variability and heterogeneity [3, 4] as well as different patterns of response to anti-VEGF treatment [5]. In Protocol T, up to half of the eyes treated were rescued with laser after 24 weeks of treatment [6]. Several attempts have been made to find markers of prognosis or predictors of treatment response in DME. The ellipsoid zone (EZ) [7], external limiting membrane (ELM) [8], disruption of the inner retinal layers (DRIL) [9], hyper-reflective retinal spots (HRS) [3], subfoveal neuroretinal detachment (SND) [4], central retinal thickness (CRT), subfoveal choroidal thickness (SFCT) [10], among others [5], have been suggested as predictors. However, some of these reports have limitations, including a retrospective profile, small sample sizes, symmetry bias and the inclusion of both Type 1 and Type 2 diabetic patients while evaluating the choroid [2].

The present study attempted to avoid those limitations and was designed to evaluate some of the predictors of outcome in eyes with recent onset DME, with special focus on SND, EZ, metabolic control, hypertension, SFCT, CRT, baseline best corrected visual acuity (BCVA), gender, duration of diabetes and history of macular laser.

## Methods

After approval from the Ethical Committees of the Faculty of Medicine of the University of Coimbra and of the Leiria Hospital, Type 2 diabetic patients with non-proliferative diabetic retinopathy (NPDR) and recent onset DME, naive to intra-vitreal treatment, were included consecutively in a prospective, institutional study, from June 2014 to June 2017. Each patient gave informed consent before inclusion in the study. The study adhered to the tenets of the Declaration of Helsinki and the standards of Good Scientific Practice of the Faculty of Medicine of the University of Coimbra. Patients were included either to be treated with ranibizumab 0.5 mg or with aflibercept 2 mg, depending on the availability of aflibercept (June 2015) and baseline BCVA according to the results at 1 year of the Protocol T study [6].

Diagnostic criteria for diabetic retinopathy (DR) and DME were based on past ophthalmic history and ophthalmic evaluation, including a dilated fundus examination, fundus photography, SD-OCT, and fluorescein angiography in selected cases.

Patient data including age, gender, blood pressure, duration of diabetes, baseline glycated hemoglobin (HbA1c) level and previous focal laser therapy were recorded.

DME was considered when clinically significant macular edema (CSME) involving the central macula (CI-CSME) or a CRT (1 mm central subfield thickness in the OCT-modified ETDRS grid) ≥300 μm was present. Eyes were included when baseline BCVA ranged from 24 to 78 ETDRS letters (L) (Snellen equivalent 20/320–20/32, LogMAR 1.22–0.14). When both eyes had DME, only one eye per patient was included [11]. The right eye was included in patients whose year of birth was an even number and the left eye was included when the year of birth was an odd number. Eyes with prior focal laser treatment were not excluded as long as laser treatment was dated more than 6 months prior to enrollment and laser burns did not involve the fovea.

Patients were excluded if they had any other previous DR treatment other than focal photocoagulation or any ocular diseases aside from NPDR in the treated eye. Eyes with a myopic refractive error of greater than 4 diopters (D) were also excluded [12]. Patients whose eyes had visually significant cataract graded at more than N03 or NC3 according to the Lens Opacity Classification Scheme were excluded.

Follow-up included baseline, 3 months (3 M) and 6 months (6 M). BCVA was measured at every visit using the ETDRS standardized scale at 4 m distance. Top score allowed for records in the ETDRS scale was 85 L (Snellen 20/20). Patients were treated with a monthly 3 injections’ loading dose and on a pro re nata (PRN) regimen thereafter.

### Imaging

EDI SD OCT (Spectralis; Heidelberg Engineering, Heidelberg, Germany) scans were performed monthly in all eyes included and guided PRN decision to treat, after the loading dose. For each study eye, a 6 mm × 6 mm macular cube scan was performed using the high resolution (HR) posterior pole scanning mode comprising 61 horizontal B-scans, 120 μm apart from each other, each made up of 1536 A-scans and a 6-line star scan, each made up of 1536 A scans, 30° apart from each other cutting through the fovea. Two independent raters (AC, do Carmo) measured all scans in a prospective way and reviewed all of them at the end of the study being masked to the subjects’ outcomes, and definitive measures were reached by consensus.

**Fig. 1:**
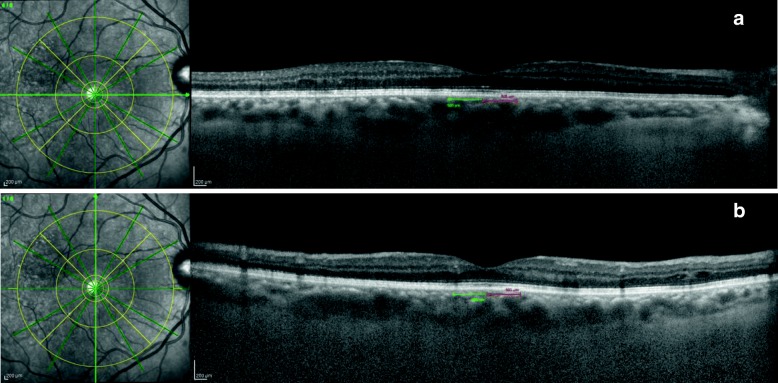
Re-rating the ellipsoid zone (EZ) after 3 injections of anti-VEGF. ETDRS grid from the caliper tool set in place centered at the fovea. **a.** Horizontal scan, 500 μm each side of the fovea to evaluate the EZ. Note that laser dots are outside the 1500 μm radius (second circle of the ETDRS grid has a radius of 1750 μm) from the foveola. **b.** ETDRS grid set in place centered at the fovea. Vertical scan, 500 μm each side of the fovea to evaluate the EZ

The average thickness of the central 1 mm field of the 6 mm OCT-modified ETDRS grid was used to evaluate changes in the CRT over time. The presumed fovea was considered as the region with the photoreceptor layer alone and was checked again retrospectively using the device’s automatic follow-up tool. SFCT was measured using the horizontal scan of the star scan mode centered at the fovea. Scans were evaluated by the two scorers after marking the choroid-scleral border (1 scan × rater × follow-up period). SFCT was manually measured from the hyperreflective line of the Bruch’s membrane to the hyperreflective line of the choroid-scleral interface (Additional file 1: Figure S1) using the digital caliper tool in the Heidelberg Eye Explorer software. Whenever there were doubts about the choroid-scleral border, measurements were compared with the horizontal line scan bypassing the fovea of the macular cube scan. The integrity of the EZ was evaluated at baseline and after the loading dose, in the central 500 μm in either direction of the fovea (Fig. 1a and b). The EZ was considered disrupted when there was any focal absence of the second hyperreflective band in the central 1000 μm either in the horizontal or in the vertical line scans centered at the fovea of the star scan mode and that could not be attributed to the shadowing effect of cysts or retinal vessels [13]. Whenever the raters did not agree, the ellipsoid zone was considered unreadable. All scans were performed from 9.00 a.m. to 1.00 p.m.

### Evaluation of outcome

For anatomic responders’ calculation, only baseline CRT values ≥300 μm were considered. Eyes with CSME or cysts in the central 1000 μm, but with CRT < 300 μm, were included in this study but were not considered when checking for anatomic responders. We defined anatomic responders as eyes having a 10% reduction in the baseline CRT either at 3 M (early) or at 6 M (late). Functional responders were also divided into early and late functional responders. Eyes with BCVA gains of ≥5 letters from baseline at 3 M were defined as early responders, while eyes with BCVA gains of ≥5 letters from baseline at 6 M only, were defined as late responders [5].

### Statistical analysis

Nominal data were described by absolute and relative frequencies. Quantitative data were described using the mean, standard deviation, median, minimum and maximum in the sample characterization. For other quantitative data, median, minimum and maximum were calculated but were omitted in the tables for convenience. Quantitative variables were assessed for normality with Shapiro-Wilk test. Comparisons between two measures, in different time points, of the same variable, were analyzed using the paired sample t-test or Wilcoxon test, taking normality requirements into account. Comparisons of quantitative variables between two groups were performed with t-Student or Mann-Whitney tests, as applicable. The association between categorical variables was assessed with Fisher’s test.

Linear multivariate regression models, where being an anatomical responder or being a functional responder were the dependent variables (baseline compared to 6 M), were built up using 12 predictors as independent variables: male gender, baseline BCVA < 65 L (Snellen < 20/50), intact baseline EZ, laser non-naivety, HbA1c, hypertension, baseline CRT, baseline SFCT, baseline SND, ranibizumab or aflibercept use and duration of diabetes. In both cases, the predictors were those that bore clinical significance in addition to those variables found to be statistically relevant (the criterion was *p* < 0.1). To evaluate whether laser treatment as a predictor of functional response was associated with the duration of diabetes, a Fisher’s test was employed. An interaction variable between diabetes duration and laser treatment was constructed and a logistic regression model was performed. The interaction variable was built with four different categories, which were DM ≤15 years and no laser treatment, DM > 15 years and no laser treatment, DM ≤15 years and laser treatment, DM > 15 years and laser treatment. This interaction variable entered in the regression model as a set of three dummy variables representing the last three categories described before (dummy1 = ‘DM >15 years and no laser treatment’, dummy2 = ‘DM ≤15 years and laser treatment’, dummy3 = ‘DM >15 years and laser treatment’). The assumptions of the model regarding residuals were observed as well as collinearity. Correlations between quantitative variables were assessed by computing Pearson or Spearman correlation coefficients, depending on whether normality requirements were met or not. The statistical analyses were performed on IBM SPSS Statistics 24 and on R 3.3.2. The level of significance adopted was 0.05.

## Results

**Fig. 2:**
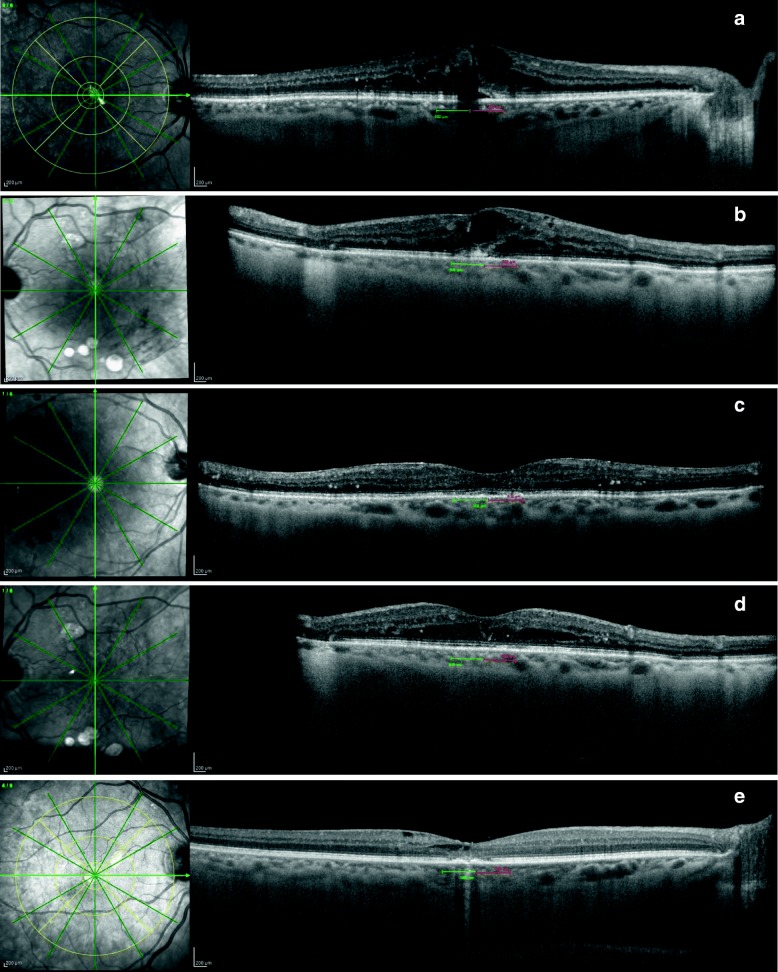
Examples of the difficulties in rating the ellipsoid zone (EZ) at baseline and after the 3-monthly injection of anti-VEGF. **a.** A small subfoveal neuroretinal detachment and in the shadowing cone effect of a retinal cyst makes the rating of the EZ difficult. In this case the EZ was rated as ‘disrupted’ by consensus. **b.** The EZ seems to be disrupted with an intact external limiting membrane (ELM). **c** and **d** Eyes shown in (a) and (b) after the loading dose. The EZ is now clearly visible, rated as ‘intact’ by both graders. **e.** EZ after the loading dose being rated as ‘disrupted’

From June 2014 to June 2017, 122 eyes from 122 patients were prospectively included and were followed for 6 M. Baseline demographic and ocular characteristics are outlined in Table 1. Baseline SND was present in 27 eyes (22.1%). Baseline EZ was intact in 80 eyes (65.5%), disrupted in 41 eyes (33.6%) and declared unreadable in 1 eye (0.8%). Graders disagreed in 14 eyes (11.1%) and a final decision was reached by consensus (Fig. 2a and b). The EZ was graded again at 3 M. It was graded as intact in 89 eyes (73.0%, Fig. 2c and d) and disrupted in 33 eyes (27.0%, Fig. 2e). At 3 M, graders agreed totally in EZ grading. The mean number of injections given was 3.0 at 3 M and 4.6 ± 1.3 (3–7) at 6 M.

**Table 1:** Demographic and ocular characteristics

Demographic characteristics (*N* = 122 patients; 68 RE and 54 LE)	
Age (years)	
Mean ± SD	65.2 ± 8.9
Median (range)	66 (46–85)
Sex	
Male	66 (54.1%)
Female	56 (45.9%)
Duration of diabetes (years)	
1–15	55 (45.1%)
16–25	52 (42.6%)
> 25	15 (12.3%)
HbA1c (%)	
≤ 7	31 (25.4%)
> 7 and < 8	44 (36.1%)
≥ 8	47 (38.5%)
Hypertension^a^	
Yes	75 (61.5%)
No	47 (38.5%)
Insulin	
Yes	64 (52.5%)
No	58 (47.5%)
Ocular characteristics (*N* = 122 eyes)	
Lens status	
Phakic	80 (65.6%)
Pseudophakic	42 (34.4%)
Intravitreal injection received	
RNZ only	93 (76.2%)
AFL only	14 (11.5%)
Both	15 (12.3%)
Laser	
Yes	68 (55.7%)
No	54 (44.3%)
Baseline BCVA (L)	
Mean ± SD	63.2 ± 12.7
Median (range)	67.0 (24.0–78.0)
Baseline CRT (μm)	
Mean ± SD	432.4 ± 107.0
Median (range)	400.5 (289.0–776.0)
Baseline SFCT (μm)	
Mean ± SD	346.6 ± 75.6
Median (range)	345.0 (124.0–580.0)
Baseline EZ	
Intact	80 (65.6%)
Disrupted	41 (33.6%)
Unreadable	1 (0.8%)
3 M EZ	
Intact	89 (73.0%)
Disrupted	33 (27.0%)
Baseline SND	
Yes	27 (22.1%)
No	95 (77.9%)

*Abbreviations*: *RE=*right eye; *LE=*left eye; *HbA1c=*level of glycated hemoglobin (percentage); *RNZ=*ranibizumab; *AFL=*aflibercept; *BCVA=*best corrected visual acuity scored using the ETDRS letters (L) chart; *CRT=*central retinal thickness; *SFCT=*subfoveal choroidal thickness; *EZ=*ellipsoid zone; *3 M EZ=*re-rating of the EZ after the loading dose; *SND=*subfoveal neuroretinal detachment; *SBP=*systolic blood pressure; *DBP=*diastolic blood pressure; *MAP=*mean arterial blood pressure

^a^SBP and DBP were measured at baseline and whenever coming back to the hospital including visits and injections. MAP was determined using the formula: MAP = DBP + 1/3 × (SBP – DBP). The patient was rated as hypertensive whenever 2 MAP values above 110 mmHg were recorded in two separate visits to the hospital

The mean baseline BCVA improved significantly, while mean baseline CRT and mean baseline SFCT decreased significantly, at 3 M and 6 M (Additional file 2: Table S1).

A total of 119 eyes out of 122 had baseline CRT values ≥300 μm and were considered for anatomic response calculation. Mean baseline CRT for a total of 98 eyes (82.4%) decreased significantly (anatomic responders) while 21 (17.6%) were non-responders. Baseline BCVA was significantly higher and CRT was significantly lower in anatomic non-responders. The mean number of injections given was also lower in the anatomic non-responders (Additional file 3: Table S2). To test whether these differences might be attributable to eyes with baseline lower CRTs and better BCVA owing to a ‘floor effect’ that decreased CRT less than 10%, we re-calculated anatomic response to include eyes with CRT above 350 μm only, to allow for a 10% decrease until 315 μm, which was previously suggested as the cut-off value for Spectralis SD-OCT [14]. Following this criterion, the differences in baseline BCVA and CRT between anatomic responders and non-responders did not stand (Additional file 4: Table S3).

A total of 74 eyes (60.7%) at 3 M and an additional 22 eyes (18.0%) at 6 M improved BCVA ≥5 L and were considered functional responders (96 eyes, 78.7%). A total of 26 eyes (21.3%) were functional non-responders. Metabolic control (HbA1c) was significantly better in functional responders (*p* = 0.003), whereas the duration of diabetes was not significantly different (*p* = 0.432) (Additional file 5: Table S4). Laser naivety was strongly associated with being a functional responder (Additional file 5: Table S4) and Table 2. Baseline intact EZ was associated with early functional response and EZ re-rating at 3 M was even more significantly associated with being a functional responder (Table 2).

**Table 2:** Comparison of OCT baseline characteristics and outcome measures between functional responders and non-responders. Baseline, 3 months and 6 months of follow-up in eyes with DME treated with anti-VEGF agents

	Functional non-responders (*N* = 26)	Early functional responders (*N* = 74)	Late functional responders (*N* = 22)	*p*-value^a^	*p*-value^b^
BCVA (L)					
Baseline	65.3 ± 10.5	62.3 ± 13.2	63.8 ± 13.4	0.535	0.447
3 M	63.9 ± 13.4	72.2 ± 11.0	65.1 ± 12.6	0.009	0.001
*p*-value 3 M	0.506	< 0.001	0.13		
6 M	64.7 ± 12.0	75.5 ± 9.6	72.7 ± 11.8	< 0.001	< 0.001
p-value 6 M	0.847	< 0.001	< 0.001		
CRT (μm)					
Baseline	420.7 ± 99.1	435.8 ± 109.5	434.9 ± 111.1	0.563	0.553
3 M	346.9 ± 90.3	334.4 ± 62.0	348.3 ± 86.7	0.712	0.792
p-value 3 M	< 0.001	< 0.001	< 0.001		
6 M	370.4 ± 111.4	332.3 ± 61.5	311.7 ± 44.4	0.455	0.460
*p*-value 6 M	0.001	< 0.001	< 0.001		
SFCT (μm)					
Baseline	339.3 ± 63.4	343.7 ± 82.6	365.3 ± 62.5	0.532	0.783
3 M	328.2 ± 71.9	319.3 ± 76.2	335.4 ± 57.5	0.746	0.597
*p*-value 3 M	0.061	< 0.001	0.003		
6 M	303.6 ± 66.4	326.9 ± 83.2	321.7 ± 66.2	0.156	0.157
*p*-value 6 M	< 0.001	0.001	< 0.001		
Baseline SND					
Yes	3 (11.5%)	21 (28.4%)	3 (13.6%)	0.187	0.111
No	23 (88.5%)	53 (71.6%)	19 (86.4%)		
Baseline EZ					
Intact	13 (50.0%)	54 (72.9%)	13 (59.1%)	0.063	0.030
Disrupted	13 (50.0%)	19 (25.7%)^c^	9 (40.9%)		
3 M EZ					
Intact	12 (46.2%)	62 (83.8%)	15 (68.2%)	0.001	< 0.001
Disrupted	14 (53.8%)	12 (16.2%)	7 (31.8%)		
Laser					
Yes	22 (84.6%)	36 (48.6%)	10 (45.5%)	< 0.001	0.001
No	4 (15.4%)	38 (51.4%)	12 (54.5%)		
Number of Injections	4.4 ± 1.3	4.7 ± 1.3	4.8 ± 1.3	0.267	0.334

*Abbreviations*: *BCVA=*best corrected visual acuity scored using the ETDRS letters (L) chart, ETDRS 62 L are Snellen 20/58, 64 L (20/53), 65 L (20/50), 72 L (20/36), 73 L (20/35) and 76 L (20/30); *3M=*3-month endpoint after the loading dose; *6 M=*6-month endpoint; *CRT=*1 mm central retinal thickness; *SFCT=*subfoveal choroidal thickness; *SND=*subfoveal neuroretinal detachment; *EZ=*ellipsoid zone; *3 M EZ=*re-rating of the ellipsoid zone after the loading dose

Number of injections is the mean number of injections given until 6 M

^a^Comparing responders vs. non-responders

^b^Comparing early responders vs. non-responders

^c^One eye with unreadable EZ. BCVA increased significantly only in functional responders. CRT and SFCT changes from baseline do not show a statistically significant difference between responders and non-responders displaying the poor correlation between functional response and anatomic response. An intact EZ at baseline was present in a higher proportion among functional responders and that was even more significant with the 3 M re-rating. Laser naivety was more commonly found in functional responders

### Baseline predictors for anatomic responders

According to the multivariate linear regression model, CRT and SND were found to significantly contribute as predictors. Indeed, following this criterion, the anatomic non-responders displayed a mean baseline CRT of 367.8 ± 58.2 μm, whereas the anatomic responders displayed a mean baseline CRT of 450.6 ± 108.3 μm (*p* < 0.001). SND was absent in all anatomic non-responders. The model was statistically significant (*Omnibus test* χ^2^(2) = 33.27, *p* < 0.001), the variance explained was 47%, *R*^2^ *Naguelkerke* = 0.470. To evaluate the model, a ROC analysis was performed, obtaining an area under the curve (AUC) equal to 0.913 (*p* < 0.001, CI 95% [0.861; 0.965]).

### Baseline predictors for functional responders

The multivariate linear regression model indicated low baseline BCVA, laser naivety, lower HbA1c and intact baseline EZ as strong predictors of being a functional responder (Table 3). Since laser treatment was found to be associated with the duration of diabetes (Fisher’s test, *p* = 0.045, OR = 2.150), an interaction variable between diabetes duration and laser treatment was constructed and a logistic regression model was performed. According to the new model, laser naivety was not found to be independent as a predictive factor (Additional file 6: Table S5).

**Table 3:** Results of the multivariate linear regression model obtained using twelve predictors of the increase of BCVA from baseline as independent variables

	Unstandardized Coefficients		Standardized Coefficients			95% Confidence Interval for B	
Model	B	SE	Beta	t	p	Lower Bound	Upper Bound
Constant	38.773	4.219		9.190	0.000	30.414	47.131
BCVAi (L)	− 0.386	0.056	− 0.611	−6.862	0.000	−0.497	− 0.274
Baseline EZ	4.291	1.407	0.257	3.049	0.003	1.502	7.079
Laser	−2.457	1.212	−0.155	−2.027	0.045	−4.858	−0.055
HbA1c_bin	−4.188	1.350	−0.232	−3.102	0.002	−6.862	−1.513
DM_bin	−0.960	1.193	−0.061	−0.805	0.423	−3.323	1.403
RNZ	−1.523	2.469	−0.062	−0.617	0.538	−6.415	3.368
AFL	−1.588	1.811	−0.086	−0.877	0.382	−5.176	1.999

Abbreviations: *BCVAi (L)=*baseline best corrected visual acuity in ETDRS letters; *EZ=*ellipsoid zone; *HbA1c_bin*=glycated hemoglobin level entered as a dichotomous variable (≤7 versus > 7); *DM_bin*=diabetes duration entered as a dichotomous variable (≤15 years versus >15 years); *RNZ*=ranibizumab; *AFL*=aflibercept; *B=*regression coefficient; *SE=*standard error for B

^a^Baseline BCVA: for each unit of increase in the baseline BCVA, there is an average decrease of 0.386 letters in the dependent variable (increase of BCVA after 6 months)

^b^Baseline EZ: eyes with intact EZ have an average increase of 4.291 letters in the BCVA after 6 months;

^c^Laser: eyes with history of macular photocoagulation have an average decrease of 2.457 letters in the dependent variable (increase of BCVA after 6 months);

^d^HbA1c: eyes with HbA1c > 7 have an average decrease of 4.188 letters in the dependent variable. In this model, diabetes duration and the use of ranibizumab or aflibercept were not statistically significant. The regression model obtained was statistically significant (*F*(4,116) = 14.791, *p* < 0.001) and the variables explained about 32% of the variance (R_adj = 0.315). The assumptions of the model regarding residuals were observed as well as collinearity

Considering the proportion of eyes that attained higher BCVA scores at 6 M, from the 41 eyes with disrupted baseline EZ, only 11 (26.8%) attained a BCVA ≥75 L (Snellen 20/32). However, from the 80 eyes with intact baseline EZ, 61 (76.3%) attained a BCVA ≥75 L (*p* < 0.001), a probability 9 times greater (OR = 8.8, CI 95% [3.7, 20.7]).

As expected, low baseline BCVA (< 65 L, Snellen < 20/50) suggested poor chances of attaining a high BCVA (≥75 L, Snellen ≥20/32) at 6 M. Indeed, out of the 50 eyes for which low BCVA values were observed, only 14 (28.0%) attained high BCVA scores. Furthermore, out of the 72 eyes for which BCVA values at baseline were ≥ 65 L, 58 (80.6%) attained high BCVA scores, a probability 11 times greater (OR = 10.7, CI 95% [4.6, 24.9]) to achieve a BCVA ≥75 L at 6 M.

## Discussion

The aim of this study was to evaluate markers of outcome in DME. Baseline CRT and baseline SND were predictors of anatomic response to treatment. An intact EZ, good metabolic control and lower BCVA were found to be baseline predictors of a better functional response. Moreover, laser naivety was found to be an indicator of better functional response.

Low baseline BCVA was predictive of having a large recovery (larger number of letters gained) but not of getting higher final BCVA scores. Therefore, the lower the baseline BCVA is, the better is the chance of getting a higher recovery in letters (functional response). However, due to the ‘ceiling effect’ existing in eyes with higher BCVA, a higher baseline BCVA has a smaller chance of closing a wider gap in the recovery of letters.

The presence of an intact EZ and better BCVA at baseline, were important for attaining higher final BCVA scores. The 3 M re-rating of the EZ strongly correlated with being a functional responder. It is not clear whether the improvement of the EZ at 3 M was due to re-arrangement of the photoreceptors, true neuronal regeneration or just better definition of the OCT scan. Laser naivety was found to be a predictor of better functional outcome, using the multivariate linear regression model. This is an important issue since laser was widely used in most RCTs [6]. Laser rescue seems to decrease the number of injections and CRT at a cost of a lesser gain in the BCVA [15], making the role of laser rescue questionable [16]. However, when using the logistic regression model, laser naivety was not independent from the duration of diabetes. This association of a factor that indicates better prognosis (laser naivety, using the multivariate regression model) with a factor that does not (duration of diabetes) may be attributed to the shift in DME treatment, from laser to anti-VEGFs, where laser naivety would be a real prognostic factor indeed or, on the other hand, may indicate that eyes with a history of laser photocoagulation had prior history of DME, therefore worsening the prognosis [17]. Good metabolic control was associated with being a functional responder, and it was confirmed to be an independent marker when using the linear multivariate regression model. These data enhance the importance of good metabolic control when using non-fixed regimens of treatment.

Our results partially agree with the results of a previous retrospective study in DME patients whose eyes were treated with bevacizumab, where previous macular laser was correlated with poor functional response [18].

SND and CRT were powerful markers of anatomic response. Furthermore, as previously described [19], we found a poor association between anatomic and functional response that withstood even when the milder forms of DME were withdrawn (Additional file 4: Table S3). These data correlate with the fact that SND is a marker of anatomic response only, while an intact EZ is a marker of functional response. Furthermore, SND probably is a marker of very recent or acute onset DME, particularly prone to a swift response to treatment, but the final BCVA lies beyond the resolution of the retinal edema, on photoreceptor integrity [2, 4, 13]. Similar to the study by Vujosevic et al., we also did not find SND to be a marker of functional outcome [4].

Our results do not agree with previous results that pointed baseline SFCT and CRT as predictors of outcome [10]. We found baseline SFCT lacking value as a predictor of outcome using the multivariate linear regression model and analyzed this factor in detail in a recent report [20].

A 3-monthly injections’ loading dose protocol was used, yet anatomical responders and early functional responders were evaluated at 3 M, where all eyes were treated alike. Moreover, most of the improvement in BCVA occurs until 3 M and this improvement predicts BCVA in the long term [21]. We used two different anti-VEGFs, ranibizumab and aflibercept. Available data suggest that these two drugs are mostly similar [6, 22]. According to the multivariate linear regression model, there was no difference between the two anti-VEGFs (Table 3).

The cut-off definition of DME by OCT is elusive or variable in most of trials and about 1 of 5 cases of DME may be missed if the diagnosis is supported only by OCT thickness measurements [23]. This is why we used the ETDRS funduscopic criteria of CI-CSME (hard exudates or hemorrhages within 500 μm of the fovea) to include cases of DME whose CRT was less than 300 μm. Only Type 2 diabetics were included because we wanted to check the value of SFCT as a prognostic marker and Type 1 diabetics have thicker choroids [2]. HR scan mode was used as it gives a higher quality image and allows a better visualization of the choroidoscleral border and of the EZ.

ELM was not evaluated, since it largely parallels the prognostic profile of the EZ [8]. Other possible prognostic markers such as cysts and DRIL were not evaluated mainly because of the limitation in the input imposed by the multivariate linear regression models. However, a recent study by one of the authors of this study compared those factors and the EZ and concluded that an intact EZ was the most reliable OCT marker of them all [24].

The biggest limitation of this study is that it is not a randomized controlled trial, does not involve multiple centers, and that factors such as DRIL, HRS or cysts were not evaluated.

The strengths of this study are related to the fact that it is a real-world study with a prospective profile, including one eye per patient only, to avoid a Type 1 error [25], the inclusion of Type 2 diabetics only, the use of the HR scan mode and the use of the ETDRS charts to evaluate BCVA.

## Conclusion

### What was known before

An intact EZ and lower baseline BCVA are predictors of functional outcome and higher baseline CRT is a predictor of anatomic outcome.

### What this study adds

SND is a predictor of anatomic outcome but does not predict the functional outcome. Neither CRT nor SFCT are predictors of functional outcome. Good metabolic control is a predictor of functional outcome in non-fixed regimens. Laser naivety is associated with being a functional responder but needs further research since it did not prove to be an independent predictor in the logistic regression model.

## Additional files

Additional file 1: **Figure S1.** ETDRS grid in place centered at the fovea. Note that ETDRS grid plotted (7.2 mm in diameter) is larger than the OCT-modified ETDRS grid (6 mm in diameter) plotted to access central retinal thickness CRT. **a** and **b** HR horizontal scans used to measure the SFCT. ETDRS grid inner circle is 1200 μm (**a**) and middle circle is 3600 μm wide (**b**). **c.** HR vertical scan with SFCT measured underneath the fovea. (DOCX 798 kb)

Additional file 2: **Table S1.** Baseline values for BCVA, CRT and SFCT. Differences in BCVA, CRT and SFCT between endpoints and baseline, and number of injections given. (DOCX 14 kb)

Additional file 3: **Table S2.** Comparison of outcome measures between anatomic responders and non-responders at baseline, 3 months and 6 months. (DOCX 17 kb)

Additional file 4: **Table S3.** Comparison of outcome measures between anatomic responders and non-responders using a cut-off for CRT of 350 μm. (DOCX 15 kb)

Additional file 5: **Table S4.** Demographic characteristics of functional responders and non-responders. (DOCX 16 kb)

Additional file 6: **Table S5.** Results of the linear regression model obtained using all predictors of the increase of BCVA as independent variables and taking into account the interaction between duration of diabetes and laser treatment found by the Fisher’s test. (DOCX 16 kb)
