# Supplementary material for: Evaluation of markers of outcome in real-world treatment of diabetic macular edema
Source: Eye Vis (Lond). 2018 Oct 11;5:27. doi: 10.1186/s40662-018-0119-9 (PMC6198537; doi:10.1186/s40662-018-0119-9)
Supplement: Supplementary file 1 — Figure S1. ETDRS grid in place centered at the fovea. Note that ETDRS grid plotted (7.2 mm in diameter) is larger than the OCT-modified ETDRS grid (6 mm in diameter) plotted to access central retinal thickness CRT. a and b HR horizontal scans used to measure the SFCT. ETDRS grid inner circle is 1200 μm (a) and middle circle is 3600 μm wide (b). c. HR vertical scan with SFCT measured underneath the fovea. (DOCX 798 kb) [file 40662_2018_119_MOESM1_ESM.docx]

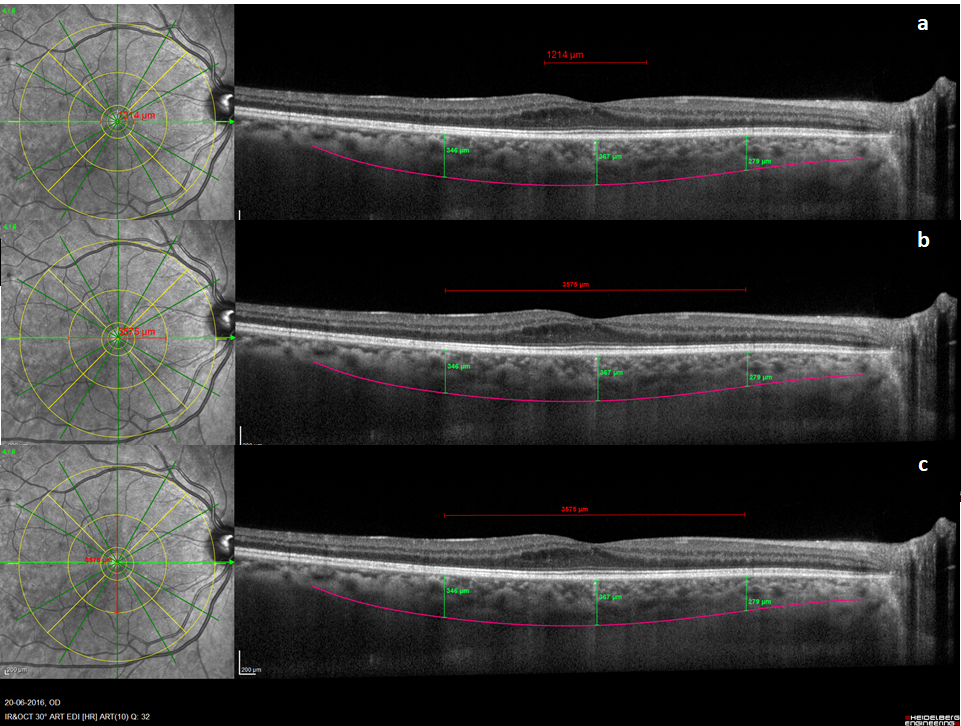


**Additional file 1: Figure S1.** ETDRS grid in place centered at the fovea. Note that ETDRS grid plotted (7.2 mm in diameter) is larger than the OCT-modified ETDRS grid (6 mm in diameter) plotted to access central retinal thickness CRT. **a.** and **b.** HR horizontal scans used to measure the SFCT. ETDRS grid inner circle is 1200 μm (**a**) and middle circle is 3600 μm wide (**b**). **c.** HR vertical scan with SFCT measured underneath the fovea.
