# Supplementary material for: Evaluation of markers of outcome in real-world treatment of diabetic macular edema
Source: Eye Vis (Lond). 2018 Oct 11;5:27. doi: 10.1186/s40662-018-0119-9 (PMC6198537; doi:10.1186/s40662-018-0119-9)
Supplement: Supplementary file 2 — Table S1. Baseline values for BCVA, CRT and SFCT. Differences in BCVA, CRT and SFCT between endpoints and baseline, and number of injections given. (DOCX 14 kb) [file 40662_2018_119_MOESM2_ESM.docx]

**Additional file 2: Table S1.** Baseline values for BCVA, CRT and SFCT. Differences in BCVA, CRT and SFCT between endpoints and baseline, and number of injections given.

|  | Baseline  (N=122) | 3M - Baseline  (N=122) | 6M - Baseline  (N=122) |
| --- | --- | --- | --- |
|  | 63.2 ± 12.7 | 5.9 ± 7.1 | 9.5 ± 7.9 |
| BCVA (L) |  | <0.001 | <0.001 |
|  |  | 60.6%^a^ | 77.9%^a^ |
|  | 432.4 ± 107.0 | -92.8 ± 103.9 | -95.7 ± 108.6 |
| CRT (µm) |  | <0.001 | <0.001 |
|  | 346.6 ± 75.6 | -22.5 ± 35.8 | -25.6 ± 44.8 |
| SFCT (µm) |  | <0.001 | <0.001 |
| N Injections |  | 3.0 ± 0.0  (3.0–3.0) | 4.6 ± 1.3  (3.0–7.0) |

Abbreviations: BCVA (L) = best corrected visual acuity scored using the ETDRS letters (L) chart: 63L are equivalent to LogMAR 0.44 or Snellen 20/55; CRT = 1 mm central retinal thickness; SFCT= subfoveal choroidal thickness; N injections = number of intra-vitreal injections given; 3M = after the 3-monthly injection loading dose; 6M = 6 months; N injections = number of injections given at each endpoint. Results are presented as mean ± SD and range for injections. ^a^Proportion of eyes that displayed an increase of 5L or more when compared to the baseline.
