# Supplementary material for: Evaluation of markers of outcome in real-world treatment of diabetic macular edema
Source: Eye Vis (Lond). 2018 Oct 11;5:27. doi: 10.1186/s40662-018-0119-9 (PMC6198537; doi:10.1186/s40662-018-0119-9)
Supplement: Supplementary file 3 — Table S2. Comparison of outcome measures between anatomic responders and non-responders at baseline, 3 months and 6 months. (DOCX 17 kb) [file 40662_2018_119_MOESM3_ESM.docx]

| **Additional file 3: Table S2.** Comparison of outcome measures between anatomic responders and non-responders at baseline, 3 months and 6 months. | | | |
| --- | --- | --- | --- |
|  | Anatomic non-responders  (n = 21) | Anatomic responders  (n = 98) | p-value |
| BCVA (L) |  |  |  |
| Baseline | 67.4 ± 11.1 | 61.9 ± 12.9 | 0.037 |
| 3M | 72.7 ± 11.5 | 68.2 ± 12.6 | 0.088 |
| 6M | 75.7 ± 10.7 | 71.9 ± 11.6 | 0.109 |
| Percentage increasing ≥5L | 61.9% | 62.2% | 1.000 |
| p-value 3M | <0.001 | <0.001 |  |
| p-value 6M | <0.001 | <0.001 |  |
| CRT (µm) |  |  |  |
| Baseline | 367.8 ± 58.2 | 450.6 ± 108.3 | <0.001 |
| 3M | 366.2 ± 71.8 | 336.4 ± 72.3 | 0.026 |
| 6M | 367.2 ± 71.2 | 332.1 ± 74.3 |  |
| p-value 3M | 0.832 | <0.001 |  |
| p-value 6M | 0.941 | <0.001 |  |
| SFCT (µm) |  |  |  |
| Baseline | 350.6 ± 73.7 | 347.3 ± 76.1 | 0.859 |
| 3M | 322.9 ± 72.0 | 326.0 ± 82.3 | 0.858 |
| 6M | 326.7 ± 70.6 | 321.0 ± 78.9 | 0.762 |
| p-value 3M | 0.003 | <0.001 |  |
| p-value 6M | 0.036 | <0.001 |  |
| Baseline SND |  |  |  |
| Yes | 0 (0.0%) | 27 (27.6 %) | 0.003 |
| No | 21 (100%) | 71 (72.4 %) |  |
| Baseline EZ |  |  |  |
| Intact | 18 (85.7%) | 59 (60.8%) | 0.042 |
| Disrupted | 3 (14.3%) | 38 (39.2%) |  |
| Laser |  |  |  |
| Yes | 14 (66.7%) | 51 (52.0%) | 0.239 |
| No | 7 (33.3%) | 47 (48.0%) |  |
| Number of injections | 4.0 ± 1.2 | 4.8 ± 1.3 | 0.016 |
| Abbreviations: BCVA = best corrected visual acuity scored using the ETDRS letters (L) chart: 62L are Snellen 20/58, 67L (20/46), 68L (20/44), 72L (20/36), 73L (20/35) and 76L (20/30); 3M = 3 month endpoint after the loading dose; 6M = 6 month endpoint; CRT = 1 mm central retinal thickness; SFCT = subfoveal choroidal thickness; SND = subfoveal neuroretinal detachment; EZ = ellipsoid zone. For anatomic responders’ calculation, only eyes with baseline CRT ≥300 μm were considered, N = 119 eyes. Eyes were considered responders if they had a 10% decrease from baseline CRT. The difference in vision gain between anatomic responders and non-responders was not statistically significant. A higher mean baseline CRT and baseline SND correlated with anatomic response. The mean baseline SFCT decreased significantly with treatment in both groups with no statistically significant difference between them. | | | |
