# Supplementary material for: Evaluation of markers of outcome in real-world treatment of diabetic macular edema
Source: Eye Vis (Lond). 2018 Oct 11;5:27. doi: 10.1186/s40662-018-0119-9 (PMC6198537; doi:10.1186/s40662-018-0119-9)
Supplement: Supplementary file 4 — Table S3. Comparison of outcome measures between anatomic responders and non-responders using a cut-off for CRT of 350 μm. (DOCX 15 kb) [file 40662_2018_119_MOESM4_ESM.docx]

| **Additional file 4: Table S3.** Comparison of outcome measures between anatomic responders and non-responders using a cut-off for CRT of 350 μm. | | | |
| --- | --- | --- | --- |
|  | Anatomic non-responders  (N=10) | Anatomic responders  (N=84) | p-value |
| BCVA (L) |  |  |  |
| Baseline | 63.0 ± 13.5 | 60.2 ± 13.0 | 0.450 |
| 3M | 69.3 ± 14.8 | 67.1 ± 12.2 | 0.407 |
| 6M | 71.7 ± 14.0 | 70.9 ± 11.6 | 0.572 |
| p-value 3M | 0.021 | <0.001 |  |
| p-value 6M | <0.001 | <0.001 |  |
| CRT (µm) |  |  |  |
| Baseline | 407.0 ± 64.3 | 471.8 ± 102.3 | 0.029 |
| 3M | 401.6 ± 80.0 | 343.8 ± 74.5 | 0.006 |
| 6M | 404.1 ± 85.8 | 340.3 ± 76.9 | 0.007 |
| p-value 3M | 0.432 | <0.001 |  |
| p-value 6M | 0.415 | <0.001 |  |
| Number of injections | 4.3 ± 1.3 | 4.9 ± 1.2 | 0.174 |
| Abbreviations: BCVA = best corrected visual acuity scored using the ETDRS letters (L) chart: 60L are Snellen 20/63, 63L (20/55), 67L (20/46), 69L (20/42), 71L (20/38), and 72L (20/36); 3M = 3-month endpoint after the loading dose; 6M = 6-month endpoint; CRT = 1 mm central retinal thickness. For anatomic responders’ calculation, only eyes with baseline CRT ≥350 μm were considered, N=94 eyes. Unlike the former criterion using a CRT cut-off of 300 μm for calculating anatomic outcome displayed in Supplementary Table 2, the differences in the number of injections given and in the baseline BCVA did not withstand with a CRT cut-off of 350 μm. Only baseline CRT was significantly higher in the anatomic responders. | | | |
