# Supplementary material for: Evaluation of markers of outcome in real-world treatment of diabetic macular edema
Source: Eye Vis (Lond). 2018 Oct 11;5:27. doi: 10.1186/s40662-018-0119-9 (PMC6198537; doi:10.1186/s40662-018-0119-9)
Supplement: Supplementary file 5 — Table S4. Demographic characteristics of functional responders and non-responders. (DOCX 16 kb) [file 40662_2018_119_MOESM5_ESM.docx]

| **Additional file5: Table S4.** Demographic characteristics of functional responders and non-responders. | | | | |
| --- | --- | --- | --- | --- |
|  | Functional  non-responders (N=26) | | Functional responders  (N=96) | p-value |
| Age (years) | |  |  |  |
| Mean ± SD | | 64.3 ± 8.6 | 65.4 ± 9.0 | 0.562 |
| Median (range) | | 66 (47-78) | 66 (46–85) |  |
| Sex | |  |  |  |
| Male | | 11 (42.3%) | 55 (7.3%) | 0.190 |
| Female | | 15 (57.7%) | 41 (42.7%) |  |
| Duration of diabetes (years) | |  |  |  |
| 1-15 | | 9 (34.6%) | 46 (47.9%) | 0.432 |
| 16-25 | | 14 (53.9%) | 38 (39.6%) |  |
| >25 | | 3 (11.5%) | 12 (12.5%) |  |
| HbA1c (%) | |  |  |  |
| <7 | | 1 (3.8%) | 30 (31.3%) | 0.003 |
| >7 and <8 | | 15 (57.7%) | 29 (30.2%) |  |
| >8 | | 10 (38.5%) | 37 (38.5%) |  |
| Hypertension^a^ | |  |  |  |
| Yes | | 19 (73.1%) | 56 (58.3%) | 0.256 |
| No | | 7 (26.9%) | 40 (41.7%) |  |
| Insulin | |  |  |  |
| Yes | | 15 (57.7%) | 49 (51.0%) | 0.659 |
| No | | 11 (42.3%) | 47 (49.0%) |  |
| Laser | |  |  |  |
| Yes | | 22 (84.6%) | 46 (47.9%) | <0.001 |
| No | | 4 (15.4%) | 50 (52.1%) |  |
| Abbreviations: HbA1c = level of glycated hemoglobin (percentage); SBP = systolic blood pressure; DBP = diastolic blood pressure; MAP = mean arterial blood pressure. MAP was determined using the formula: MAP = DBP + 1/3 × (SBP - DBP). ^a^The patient was rated as hypertensive whenever two MAP values above 110 mmHg were recorded in two separate visits to the hospital. Baseline demographic characteristics show a statistically significant difference between functional responders and non-responders for metabolic control and laser treatment. Interestingly, the duration of diabetes is not a factor influencing the prognosis when defining functional response as a gain of 5 letters. | | | | |
