# Supplementary material for: Evaluation of markers of outcome in real-world treatment of diabetic macular edema
Source: Eye Vis (Lond). 2018 Oct 11;5:27. doi: 10.1186/s40662-018-0119-9 (PMC6198537; doi:10.1186/s40662-018-0119-9)
Supplement: Supplementary file 6 — Table S5. Results of the linear regression model obtained using all predictors of the increase of BCVA as independent variables and taking into account the interaction between duration of diabetes and laser treatment found by the Fisher’s test. (DOCX 16 kb) [file 40662_2018_119_MOESM6_ESM.docx]

| **Additional file 6: Table S5.** Results of the linear regression model obtained using all predictors of the increase of BCVA as independent variables and taking into account the interaction between duration of diabetes and laser treatment found by the Fisher’s test. | | | | |
| --- | --- | --- | --- | --- |
| Predictor (reference) | B | SE | p-value | 95% CI for B |
| Baseline BCVA (L) | -0.397 | 0.055 | <0.001 | (-0.505, -0.289) |
| Baseline EZ | 4.917 | 1.453 | 0.001 | (2.040, 7.795) |
| Dummy 1 | 1.136 | 1.808 | 0.531 | (-2.444, 4.717) |
| Dummy 2 | -0.936 | 1.794 | 0.603 | (-4.490, 2.617) |
| Dummy 3 | -3.591 | 1.567 | 0.024 | (-6.694, -0.488) |
| Constant | 32.590 | 3.247 | <0.001 | (26.158, 39.022) |
| Abbreviations: BCVA (L)= best corrected visual acuity scored using the ETDRS letters (L) chart; EZ = ellipsoid zone; B = regression coefficient; SE = standard error for B; 95% CI for B is the 95% confidence interval for the regression coefficient; DM = duration of diabetes. The interaction variable between diabetes duration and laser treatment has four different categories: DM ≤15 years and no laser treatment; DM >15 years and no laser treatment; DM ≤15 years and laser treatment; DM >15 years and laser treatment. This interaction variable entered in the regression model as a set of three dummy variables representing the last three categories described before (Dummy 1 = DM >15 years and no laser treatment; Dummy 2 = DM ≤15 years and laser treatment; Dummy 3 = DM >15 years and laser treatment). In this model, the variables Dummy 1 and Dummy 2 were not statistically significant. The meaning of the regression coefficient is similar to the first model (Supplementary Table 5) except for the variable Dummy 3. In this case, the B value means that eyes of patients being diabetic for more than 15 years and undergone laser treatment present on average a decrease of 3.591 letters in the dependent variable (increase of BCVA after 6 months) when compared to the eyes of patients that did not have laser treatment and have diabetes for less than 16 years. The model attained was statistically significant (F(5,115) = 12.624, p<0.001) and the variables explained about 33% of the variance (R_adj = 0.326). The assumptions of the model regarding residuals were observed as well as collinearity. | | | | |
